# *Mycoplasma bovis* in Nordic European Countries: Emergence and Dominance of a New Clone

**DOI:** 10.3390/pathogens9110875

**Published:** 2020-10-23

**Authors:** Florence Tardy, Anna Aspan, Tiina Autio, Anne Ridley, Agnès Tricot, Adélie Colin, Tarja Pohjanvirta, Bregtje Smid, Frank Harders, Mikkel Lindegaard, Klara Tølbøll Lauritsen, Ulrike Lyhs, Henk J. Wisselink, Mikael Lenz Strube

**Affiliations:** 1 UMR Mycoplasmoses des Ruminants, ANSES laboratoire de Lyon, VetAgro Sup, Université de Lyon, 69364 Lyon, France; agnes.tricot@vetagro-sup.fr (A.T.); adelie.colin@anses.fr (A.C.); 2 National Veterinary Institute (SVA), SE-751 89 Uppsala, Sweden; anna.aspan@sva.se; 3 Finnish Food Authority, 70210 Kuopio, Finland; tiina.autio@ruokavirasto.fi (T.A.); tarja.pohjanvirta@ruokavirasto.fi (T.P.); 4 Animal and Plant Health Agency (APHA), Surrey KT15 3NB, UK; Anne.Ridley@apha.gov.uk; 5 Wageningen Bioveterinary Research, 8221 RA Lelystad, The Netherlands; Bregtje.smid@wur.nl (B.S.); frank.harders@wur.nl (F.H.); henk.wisselink@wur.nl (H.J.W.); 6 National Veterinary Institute, Technical University of Denmark, 2800 Kgs Lyngby, Denmark; ml@mixs.dk (M.L.); klaratl@gmail.com (K.T.L.); ulrike.lyhs@helsinki.fi (U.L.)

**Keywords:** cattle, antimicrobial resistance, genomic epidemiology, phylogenetics, *Mycoplasma bovis*

## Abstract

*Mycoplasma* (*M*.) *bovis* is an important pathogen of cattle implicated in a broad range of clinical manifestations that adversely impacts livestock production worldwide. In the absence of a safe, effective, commercial vaccine in Europe, reduced susceptibility to reported antimicrobials for this organism has contributed to difficulties in controlling infection. Despite global presence, some countries have only recently experienced outbreaks of this pathogen. In the present study, *M. bovis* isolates collected in Denmark between 1981 and 2016 were characterized to determine (i) genetic diversity and phylogenetic relationships using whole genome sequencing and various sequence-based typing methods and (ii) patterns of antimicrobial resistance compared to other European isolates. The *M. bovis* population in Denmark was found to be highly homogeneous genomically and with respect to the antimicrobial resistance profile. Previously dominated by an old genotype shared by many other countries (ST17 in the PubMLST legacy scheme), a new predominant type represented by ST94-*adh1* has emerged. The same clone is also found in Sweden and Finland, where *M. bovis* introduction is more recent. Although retrieved from the Netherlands, it appears absent from France, two countries with a long history of *M. bovis* infection where the *M. bovis* population is more diverse.

## 1. Introduction

*Mycoplasma (M.) bovis* is one of the currently described more than 130 species of the bacterial genus *Mycoplasma* and one of the major causative agents of bovine mycoplasmosis [1]. It was first reported in a case of mastitis in 1961 in the USA [2] and since then has been increasingly associated with a broad range of clinical manifestations, such as pneumonia (as one of the etiological agents of the bovine respiratory disease, BRD), arthritis, otitis media, and genital disorders. It is distributed worldwide, with variable prevalence, and considered to be one of the major emerging pathogens of cattle in industrialized countries threatening livestock production [3] and accounting for significant economic and production losses in the beef and dairy industries [4]. The recent introduction of the disease in New Zealand (https://www.mpi.govt.nz/protection-and-response/mycoplasma-bovis/) and Finland [5] prompted the establishment of drastic eradication measures or control programs. The growing concern of farmers and veterinarians over the last decades has been supported by several scientific studies aimed at deciphering epidemiological trends, antimicrobial resistance, pathogenesis, isolate diversity and spread, as well as diagnostics optimization. Despite these efforts, extensive knowledge gaps regarding *M. bovis* disease, prevention, and control still remain [1]. For instance, there is still no efficient commercial vaccine available against *M. bovis* in Europe. Moreover, few studies on the performance of the vaccines currently licensed in the USA exist (for reviews see [1,6]), while autogenous vaccines generally lack an evidence base for their efficacy. Vaccine development is vital as several studies have highlighted the in vitro resistance of *M. bovis* to most of the antimicrobial families currently in use, except fluoroquinolones [7]. However, the application of the later class of antimicrobials is now restricted in food-producing animals because of their importance in human medicine (e.g., https://www.oie.int/en/for-the-media/amr/oie-amr-standards/).

Several whole genome sequencing (WGS) studies dedicated to *M. bovis* have been conducted for the purpose of phylogenetic typing or characterization of antimicrobial resistance determinants, but these have essentially focused on national populations [8,9,10,11]. However, as summarized by Baker et al., “microbial populations do not respect political boundaries,” and there is an urgent need for an international genomic surveillance [12]. One issue within the *Mycoplasma* genus is that only circa 895 genomes, mostly incomplete, are currently available in the NCBI assembly database (https://www.ncbi.nlm.nih.gov/assembly/, accessed on 07 August 2020), which is remarkably few compared to other bacterial genera. Specifically, for *M. bovis*, there are currently only 269 genome assemblies available. Most of them are incomplete, including 175 derived from surveillance projects. Of the 29 fully assembled genomes, eight represent isolates from China, 16 are from a recent study in Canada, one is the species type strain isolated in the USA (PG45^T^), and only one is of European origin, JF4278, from Switzerland [13,14,15,16,17].

In Denmark, *M. bovis* was isolated for the first time in 1981, from a pneumonic calf, and by 1984, the first cases of *M. bovis* mastitis had been diagnosed [18]. From then on, the prevalence of *M. bovis* pneumonia in Danish cattle has increased and was rapidly considered a threat for dairy production. However, studies on prevalence and isolate diversity are scarce in Denmark [19,20,21]. At the end of 2011, dairy farms all over Denmark experienced a very sudden increase in severity of *M. bovis* infections with severe arthritis and diffusely swollen legs in addition to mastitis and other clinical signs in both adult and young dairy cattle [22]. Finland and Sweden have also experienced emergence, but only over the last 7 to 8 years, while Norway is still considered free of *M. bovis* despite the lack of recent publicly available data [5,23]. In comparison, the disease has been prevalent for more than 40 years in other European countries, such as France [24,25], where characterization tools have been used to investigate the *M. bovis* strain population. Extending our understanding of the evolutionary history and population dynamics of *M. bovis* to the isolates present in Nordic European countries is essential for the development of appropriate and widely applicable control measures and diagnostic tools adapted to the disease epidemiology.

The present study aimed at analyzing the genetic relatedness and evolution of 73 *M. bovis* clinical isolates collected in Denmark between 1981 and 2016, including several from the aforementioned 2011 outbreak, compared to other isolates representative of the epidemic situation in other Nordic countries (Finland and Sweden, n = 3) or other European countries (France, the Netherlands, or Estonia, n = 21). For that purpose, the genomes of the individual isolates were sequenced and compared taking account of their individual history (mainly, year, country, and body site of isolation). We also included four high quality genomes of representative strains as references. Genome-wide methods were compared to the previously established, as well as newly proposed, multilocus sequence typing (MLST) schemes [26,27,28] to explore the congruence of the population structure and compare it with the isolates circulating elsewhere in the world. Additionally, the diversity of our *M. bovis* study population was assessed in terms of susceptibility to the commonly used veterinary antibiotics in order to assess the potential for antibiotic resistance lineages in Nordic European countries, from where data are scarce.

## 2. Results

### 2.1. Genetic Structure of Our M. bovis Population

Ninety-seven *M. bovis* isolates were sequenced, of which 73 were from Denmark (Supplementary Table S1). They originated from the respiratory tract (lung / nasal swabs / bronchoalveolar fluids, n = 32), joints (n = 33), or other body sites (n = 13; ear canal, eye, semen, or milk). The site of origin was unknown for 19 Danish isolates. The vast majority of the isolates from sampling of joints associated with arthritis (32/33) were collected in Denmark between 2011 and 2015 [22]. The isolates collected between 1981 and 2000 in Denmark (n = 26) are for the purposes of this study hereafter referred to as old isolates. One isolate from Sweden was also included in the old category, having been isolated in 1996 from a single occurrence of an infected animal on a cattle farm (A. Aspan, personal communication). Seventy more recent isolates, all collected after 2011, were included, comprising 47 from Denmark, two from other Nordic countries (Finland and Sweden), and 23 from elsewhere in Europe for comparison.

All genomic assemblies had an N50 superior to 12,000 nucleotides (with a 17,169 median), a median coverage of 126× (range 54–927) and a median number of contigs of 99. The median number of coding sequences (CDS) was 775 (see Supplementary Table S1 for details).

**Figure 1:**
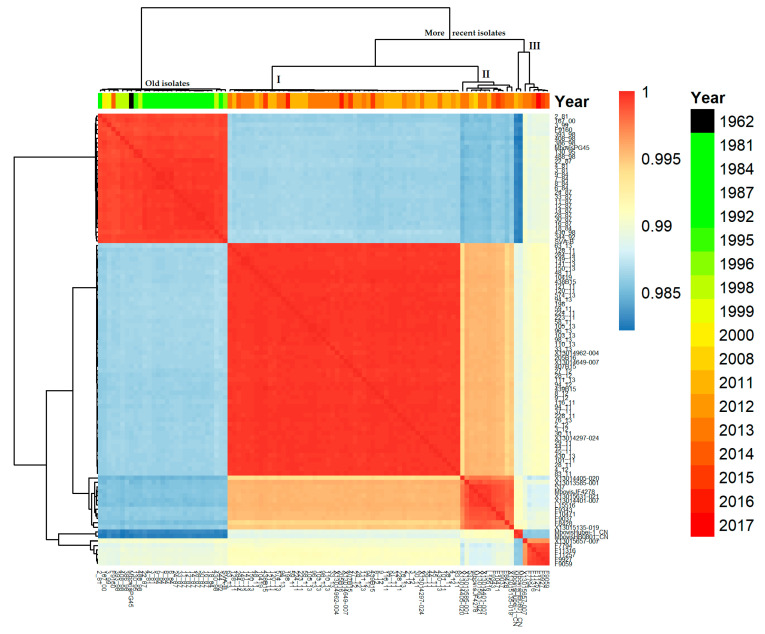
Nucleotide identity matrix for all isolates. Color values denote high (red) and lower (blue) similarity for each pairwise comparison. Clusters are based on complete linkages.

The average nucleotide identity (ANI) calculated using Pyani and the aligned genome sequences revealed a high level of similarity between isolates; in all cases, this was greater than 98%, which is in the upper range of the ANI expected within a bacterial species (>95%) (Figure 1). The isolates from Denmark collected on or before 2000 were all gathered in one lineage together with type strain PG45^T^ and the old isolate from Sweden (SVA-B). Strain F9160 from France was the only exception of a recent strain grouped among the old isolates. Most of the isolates from 2011 onwards were clustered into one main branch with three distinct lineages. The first lineage was highly homogeneous (lineage I, n = 52) and contained all recent Danish isolates together with the more recent Finnish and Swedish strains, as well as three strains from the Netherlands. The second lineage (lineage II, n = 12) was more heterogeneous and comprised French (n = 5) and Dutch (n = 5) isolates, together with Swiss strain JF4278 from 2008 and a single Estonian isolate. The remaining lineage includes five French isolates and one from the Netherlands, together with the Chinese reference strains, which are almost 2% dissimilar from their nearest neighbor (Figure 1). Clustering of isolates was further analyzed using two other WGS-based methods, a core-genome MLST (cgMLST) approach, and an SNP (Single-Nucleotide Polymorphism)-calling approach. 

**Figure 2:**
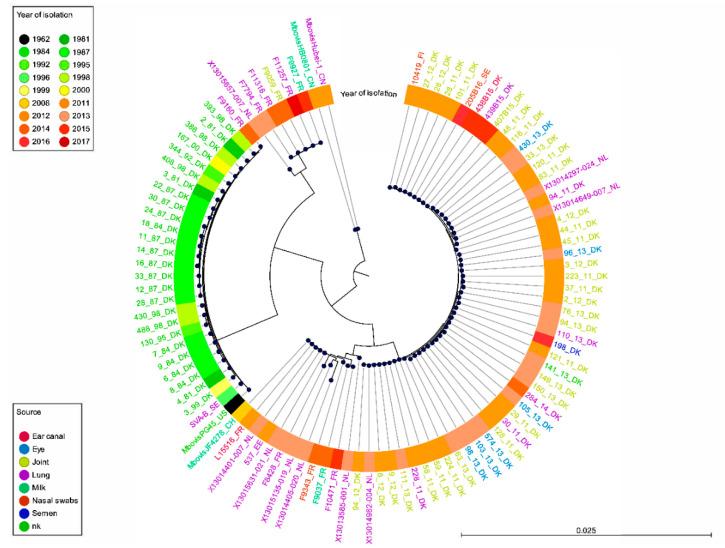
Phylogenetic tree based on cgMLST as generated by FastTree and stratified by body site of isolation and country and labeled by year of isolation. The PG45 type strain from the USA as well as two Chinese isolates (Chinese cluster) and the recent Swiss isolate, JF4278, were included for comparison. The Chinese Hubei-1 isolate was used as an outgroup. The core genome was generated by Roary and was constituted of 500 genes out of a total of 1807 genes. nk, not known.

The cgMLST analysis was based on the alignment of 500 core genes, out of a pan-genome of 1807 genes, using the open reading frames that were more than 95% identical in 99% of the isolates. This alignment was used to infer a phylogenetic tree using the two Chinese reference strains as an outgroup as they were more remote in the ANI analysis (Figure 2). The resulting pattern confirmed the split between the old and the more recent populations, with the exception of the recent F9160 isolate which was grouped in the set of old isolates like in the ANI analysis. Recent isolates from Nordic countries (as well as three isolates from the Netherlands) once again showed homogeneity, whereas more variability was observed between isolates from other countries, with some new clusters (sub-branches) appearing. These other sub-branches comprised isolates from France, the Netherlands, Estonia and the Swiss reference strain JF4278. One was closer to the group of old isolates (five French isolates and one Dutch isolate), the other branched with new Nordic isolates. This distribution was consistent with that of the ANI analysis, with a group of recent strains from France closer to the old isolates group. All branches and sub-branches contained a mix of strains isolated from different sites in the body, with no evidence to suggest anatomical specificity of strains. As an alternative to cgMLST, which took into consideration the genes conserved in all genomes (n = 500 here), a genome-wide SNP phylogeny [29] was also generated on our set of genomes using the methodology already validated for *M. bovis* [11]. The resulting phylogenetic tree corresponded well to that of the cgMLST (Figure S1), showing the same phylogenetic signals, albeit with a lower discriminatory power.

All these isolates distribution patterns are suggestive of (i) a genomic shift in the Danish population structure with time and also of (ii) a potential divergent evolution of isolates after the year 2008, with isolates from Nordic countries clustered in a separate branch and more diversity for the isolates elsewhere in Europe. To further investigate this hypothesis of clonal spread in Nordic countries, MLST analyses were performed to facilitate comparison of an enlarged set of isolates that better represents the global *M. bovis* population worldwide.

### 2.2. Studying of the M. bovis Population Structure Using Different Gene-Based Typing Schemes and an Enlarged Worldwide Set of Isolates

In order to assess their relative discriminatory ability on our set of strains and their maintained effectiveness to determine phylogenetic clusters in the genomic era, the MLST schemes from Register et al. in its legacy or new version (MLST-1a, MLST-1b, respectively) [26,28], that of Rosales et al. (MLST-2) [27], and the *polC* subtyping scheme proposed by Becker et al. [24] were used to infer the *M. bovis* population structure (see all details in Table S1).

The distribution of *polC* subtypes confirmed the split between old (st1) and more recent (st3) isolates from Denmark but was poorly discriminative for recent strains with only two subtypes identified on the whole set of strains, st2 and st3, st3 being largely dominant (Table S1). In contrast, the stratification of the cgMLST phylogeny by MLST-1a subtypes (STs) was very coherent (Figure S2), confirming its important discriminatory ability. The isolates collected before year 2001 were shown to belong to subtype ST17 (n = 25/27), like type strain PG45^T^, with two variants that differed from ST17 by one SNP in a single gene (Supplementary Table S1). Of note, all recent Danish isolates (n = 47) belonged to a variation of ST94, in which the isolates were devoid of the *adh1*-gene, with the exception of two novel variants with a different *gltX* allele (438B15 and 439B15, see Table S1). This ST94*-adh1* profile was also found in Finland, Sweden, and the Netherlands. This absence of the *adh* gene was also noted in other strains belonging to different subtypes (ST5*-adh1*, ST17*-adh1*). Other minor subtypes (ST5, 12, 14, 18, 19, 42) were found mainly in France and the Netherlands, with two recent isolates found to be variants of ST5 differing by one SNP (ST184 and ST185). The potential clonal spread of ST94-*adh1* in Denmark and its appearance in other Nordic countries contrasts with the diversity of STs in France and the Netherlands.

**Figure 3:**
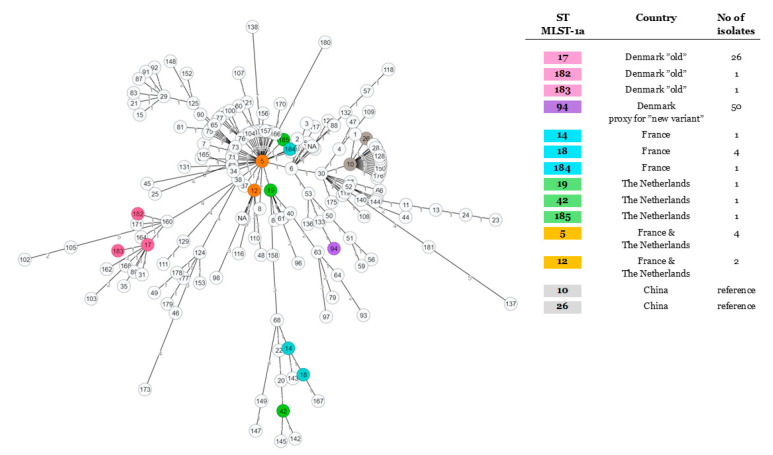
Minimum spanning tree, based on the MLST-1a from Register et al., 2015. Strains from this study are color-coded. The two isolates from China included for comparison are in grey.

The minimum spanning tree built using MLST-1a alleles and an enlarged set of strains available at https://pubmlst.org/mbovis/ showed evidence of one major cluster gathered around ST5, which could be considered as a majority “common ancestor” (Figure 3). The ST17 node gathered the strains isolated prior to 2000, the only exception to this chronological trend being a single isolate from Slovakia dating from 2012 (PubMLST) and a French strain (F9160) from the current study; in each case, the allelic profile was consistent with ST17, excepting the lack of the *adh* gene. All our recent isolates seem to radiate from ST5, either by a short (ST12, 19, 184, 185) or a greater evolutionary distance (one lineage with ST14, 18, and 42 and another one with ST94-*adh1*). Isolates from all over the world (as seen in entries from PubMLST) share this similarity to ST5, confirming a worldwide distribution. Isolates sharing STs that are more genetically distant from ST5, such as ST14, 18, and 42 (France and the Netherlands in our data set), are also noted to be geographically and temporally widespread, appearing in the US in strains from 1994 and more recently in central Europe and Israel, according to the PubMLST data. As a consequence of the absence of the *adh-1* gene, we were unable to further investigate the closest common ancestor to subtype ST94-*adh1* subtype. With the new Register scheme (MLST-1b, Figure S3), the resulting spanning tree appears similar in structure, despite comprising a more limited strain set. The MLST-1b ST29 attributed to the Nordic strains was also found in the isolates from Israel (five mastitic isolates between 2012 and 2017) as well as Hungary (one pneumonic strain isolated in 2016). ST29 seems to be derived from ST30 that notably comprises bison strains, while the closely associated ST31 and ST32 include mastitic isolates from Israel (2017) and Romania (2013), respectively. This observation confirms that the clone present in Denmark and other Nordic countries is also present outside of the Northern and Western Europe (Figure S3).

The MLST-2 scheme [27] also overcame the difficulties encountered by the absence of the *adh-1* gene of MLST-1a, facilitating type designation of the corresponding strains (Figure S4). All ST94-*adh*1 strains in MLST-1a belong to ST48 in MLST-2 or closely related newly designated variants (different shades of purple in Figure S4) radiating from ST22, a subtype identified in strain X13013585-001 from the Netherlands. Old Nordic isolates from Denmark and Sweden are clustered in the ST19 node, with old isolates from all over the world.

### 2.3. Diversity of Antimicrobial Resistance Phenotypes in the Study Population

MIC (Minimum Inhibitory Concentration) analyses were performed on a subset of 81 isolates for five widely used antimicrobials representing different classes, i.e., enrofloxacin (fluoroquinolone), oxytetracycline (tetracycline), spectinomycin (aminocyclitol), florfenicol (amphenicol), and tilmicosin (macrolides) (Table S1). These 81 isolates comprise 21 old isolates collected up to year 2000 from Denmark and 60 recent isolates (from 2011 onwards), of which 38 are Danish.

**Figure 4:**
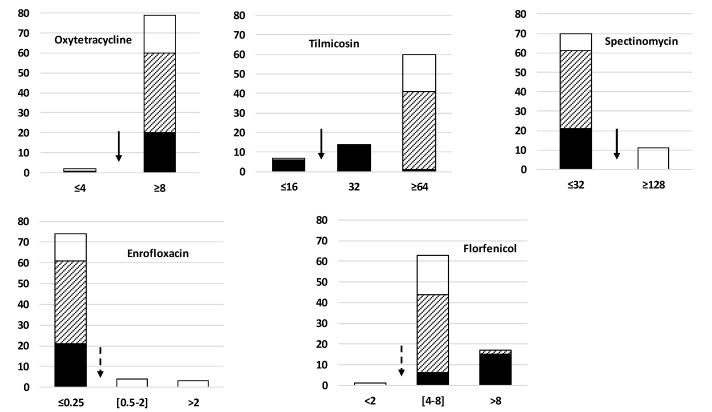
Comparison of MIC distributions of five antimicrobials for 81 *Mycoplasma bovis* isolates collected in Denmark and five other Northern and Western European countries between 1981 and 2017. X-axis, MICs in µg/mL; Y-axis, number of isolates. Black blocks represent old Danish isolates collected prior to and including 2000, striped blocks are for more recent Danish, Swedish, and Finnish isolates, with white blocks for recent isolates from Estonia, France, and the Netherlands. Arrows indicate break points for clinical categorization according to the CLSI standards for Pasteurellaceae. Any MICs above the plain arrow will categorize the strain as “Resistant”, any MICs above the dotted arrow will categorize the strain as “Intermediate or Resistant”.

As shown in Figure 4, despite the extended period for collecting the isolates (between 1981 and 2017) and their different geographical origin, their antimicrobial susceptibility distributions were notably homogeneous. Applying the CLSI (Clinical & Laboratory Standards Institute) clinical breakpoints of the Pasteurellaceae family, a group of other bacteria occupying the same body niche [30], the isolates were mostly resistant to tilmicosin, oxytetracycline, and florfenicol, but mainly susceptible to enrofloxacin and spectinomycin. Old Danish isolates collected up to year 2000 were already resistant to oxytetracycline (except one strain) and tilmicosin (except 6 strains). The atypical recent French isolate F9160 has remained susceptible to these two molecules. Interestingly, the highest MICs for florfenicol (>8µg/mL) were obtained with a majority of old isolates from Denmark (15/21), but with only 2 recent isolates (1 from Sweden and 1 from Denmark). It is noteworthy that high MIC against spectinomycin with MIC ≥ 128 µg/mL was only noticed in isolates from France (n = 9/11) and the Netherlands (n = 2/8), but in no isolates from Denmark. Similarly, all strains with increased enrofloxacin MIC values (n = 8) were from France.

As expected for ruminant mycoplasmas, no antibiotic resistance genes were found by in silico analysis of the genome. Almost all isolates had several 16S and 23S rRNA gene mutations in hotspots consistent with the observed resistance to tetracycline and macrolide (data not shown) [10,31,32].

## 3. Discussion

In this study, various comparative genomic analyses were performed on ninety-seven *M. bovis* isolates gathered from Denmark (n = 73) with a further twenty-four representing five other Northern and Western European countries compared. Old (collected in or before the year 2000) and more recent (year 2011 and after) isolates from Denmark and Sweden enabled the investigation of evolutionary trends in the *M. bovis* genomic background. The different countries participating in this study had contrasting histories with diagnosed *M. bovis* infections (see Introduction) and different livestock density, Denmark and the Netherlands featuring in the top four in Europe. Taken together, the results of all the different genome or loci-based analyses were mainly in agreement and suggest that the old clonal type closely related to the historical PG45 type strain that circulated in Denmark in the 1980s and 1990s appears to have been replaced by strains belonging to a new dominant genotype. This new dominant Danish clone might have spread to other Nordic countries that are experiencing recent emergence of *M. bovis* like Finland and Sweden and is also present in the Netherlands. In contrast, a variety of genotypes coexist in France and the Netherlands, some closer to the old isolates group.

A similar scenario of “extinction” of a dominant circulating clonal type has already been described in France, where *M. bovis* isolates belonging to *polC*_st1 have not been detected since the year 2000 and have been replaced by *polC*_st2 and *polC*_st3 that were both resistant to the most frequently used antimicrobials except fluoroquinolones. This finding is consistent with the spread of a single clone throughout the country that was hypothesized to be related to antimicrobials used as BRD treatment and restrictions to cattle trade as a consequence of the bovine spongiform encephalopathy crises [24]. The MLST-genotypes that have emerged since the year 2008 across Northern and Western Europe differ between the individual countries. For instance, the ST94-*adh1*, ST29 subtype in MLST-1a or MLST-1b, respectively, is clearly dominant in Denmark, Sweden, and Finland (even if only a single isolate from Finland and Sweden was included as representative of these countries [33]), which is consistent with the previously known spread of a single clonal type. It is also detected in the Netherlands. Elsewhere in the world, ST29 has only been detected in Israel and Hungary. Interestingly, its closest subtype (ST30) was found in bison from Canada [28]. The observation of an ST linked to bison as the closest relative of the recent Danish isolates is very interesting and warrants further attention to help elucidate the origin of these isolates now present in Europe. In France and the Netherlands, strains appear to be more genetically diverse, potentially suggesting multiple sources of entry of the circulating strains (as the corresponding MLST subtypes are largely distributed worldwide), in contrast to the more limited importation of cattle in Nordic countries. Interestingly, in France, *M. bovis* is mainly associated with respiratory disorders, with particular concern in veal fattening units, and is only diagnosed in a few cases of mastitis each year [24,32]. In the Netherlands, mastitis seems more frequent and is also of major importance in Denmark [34,35], with dairy farms all over Denmark experiencing outbreaks with increased severity, including severe arthritis and diffusely swollen legs from 2011 onward [22]. Whether this could be linked with the different genotypes currently circulating warrants further investigation. Such a hypothesis has already been proposed in Switzerland and Israel, where the shift in circulating *M. bovis* isolates in 2007 and 2008 was associated with the emergence of severe clinical mastitis cases [36,37]. Cattle trade has been put forward as a source of dissemination of *M. bovis* across the globe [11], supported here by the worldwide distribution of the strains belonging to different MLST-subtypes. Within Europe, animal movements can be traced using resources from the European Community (https://ec.europa.eu/food/animals/live_animals_en). For instance, trade between Denmark and France is extremely limited while being important between Denmark and the Netherlands, which could explain the ST94-*adh1*/ST29 presence in the Netherlands, but not in France. This observation also warrants further attention. It was also recently demonstrated that the use of contaminated semen during artificial insemination could contribute to *M. bovis* spread between farms and/or countries and could be at the origin of the clonal spread from Denmark to Finland and Sweden [33].

*M. bovis* as a species is known to be highly homogeneous. This was confirmed in the current study with a >98% overall nucleotide identity for the whole set of isolates, irrespective of country of origin, year, body site of isolation, or associated clinical manifestation as demonstrated by our ANI analysis. In this context, tools to discriminate different isolates and understand epidemiological trends are of utmost importance, but are not all equivalent. For instance, *polC* subtyping was insufficiently discriminatory for recent isolates, generating only two subtypes, with the majority of isolates assigned to st3, while st2 isolates appeared restricted to France or the Netherlands. In France, st2 is currently the main clone in the circulation, with st3 constituting a maximum of 20% of the *M. bovis* population to date [38]. The st2 and st3 subtypes were further subdivided into 16 subtypes by MLST-1a [26] (Table S1), indicating that MLSTs better inform international epidemiological trends. However, more than half of the tested isolates (n = 63) lacked the *adh-1* gene, one of the 7 loci included in this MLST-1a scheme, limiting the ability of the scheme to effectively subtype such isolates. The *adh-1* gene encodes the alcohol dehydrogenase responsible for alcohol oxidation, a characteristic known to vary between *Mycoplasma* species [39]. The absence of the *adh-1* locus was recently described in different isolates, including Swiss strain JF4278 used here as a reference [40]. Only recent isolates were characterized by the loss of the *adh-1* gene and they belong to three different MLST-subtypes (ST5, ST17, and ST94 in MLST-1a) suggesting this feature may be a consequence of evolutionary genome reduction associated with host adaption described in mycoplasmas, apparently irrespective of the genomic background. The issue of being unable to designate types for MLST-1a was recently addressed in an updated MLST scheme, which uses *dnaA* instead of *adh-1* [28] (see https://pubmlst.org/mbovis/). It already comprises 464 entries and was shown to have a better discriminatory index than the “legacy” base or than the MLST scheme from Rosales et al. (MLST-2 here) [27]. However, the *dnaA* allele alone was poorly discriminatory for the isolates included in our study. Because it considers evolutionary signals originating from the whole genome, either the whole set of CDS (cgMLST) or genome-wide polymorphisms, the WGS-typing approaches are a more appropriate way forward [29]. However, they remain relatively expensive, time-consuming, and require expertise for interpretation. Hence, for *M. bovis*, the existence of the pubmlst.org database remains an asset for the whole scientific community. This study has contributed to the public database (both the genomic and MLST databases) by including a wider range of isolates from Europe. For instance, previously, only 33 isolates in the PubMLST set of 448 isolates studied by Register et al. were of European origin [28]. We have been able to include older isolates, collected prior to 2000, that are currently less well represented in the MLST scheme and genomic data.

Understanding the epidemiology of these old isolates is undoubtedly important. One current genotype (or subtype) in France (strain F9160) clustered with old isolates, suggesting it could result from a re-emergence of an old clone that had continued to circulate for years in an unusual host or in an environmental reservoir. The F9160 isolate was isolated in 2014 from a calf in a dairy herd located in the Southwest of France, a region mainly known for ovine breeding. In common with recent strains, the *adh-1* gene was absent, a characteristic not previously described in ST17 of MLST-1a, suggesting the loss of this gene may be an evolutionary trait shared by different phylotypes.

In France, the apparent disappearance of the *polC*-st1 (ST17 in MLST-1a) and the emergence of two new subtypes since 2000 has been associated with a loss of susceptibility to antimicrobials [24]. Here, most of the current strains, whatever their country of origin, and their genotypes would be classified as resistant using the clinical breakpoints of the Pasteurellaceae family, except for fluoroquinolones and spectinomycin. For compounds such as oxytetracycline and tilmicosin, most of the old Danish isolates collected in the 1980s or 90s were already resistant, which is consistent with the date these now commonly used antimicrobials were originally marketed, as already observed in France [32]. In contrast, for florfenicol, the old Danish isolates appeared less susceptible than the recent ones. This observation appears unrelated to the change in use, as the DANMAP2018 reports stated that the florfenicol usage has increased in Denmark in the last decade (https://www.danmap.org/-/media/arkiv/projekt-sites/danmap/danmap-reports/danmap-2018/danmap_2018.pdf?la=en). Loss of susceptibility to spectinomycin was only noticed in isolates from France (n = 12/14) and the Netherlands (n = 2/9) as noted earlier [34,41]. Similarly, all strains with increased enrofloxacin MIC values (n = 9) were from France despite being classified as critical for human medicine and, presumably, its use restricted in food-producing animals. These few differences in antimicrobial susceptibility might be the consequence of different antimicrobial usage in different countries, or the influence of the genomic background on the capacity to acquire resistance. This influence has already been demonstrated in vitro that *M. bovis* strains of st3 in France were more prone to acquiring and fixing mutations in the quinolone resistance-determining regions than those belonging to st2 [31]. Interestingly, for spectinomycin, to date, the mechanisms associated with resistance phenotypes are still unknown. The clone currently spreading in Nordic countries is in vitro multiresistant, but spectinomycin remains an option for treatment.

In contrast, French strain F9160, which shares its genomic background and genetic subtypes with the strains in Denmark isolated prior to 2000, correspondingly features high susceptibility to the tested antimicrobials. This observation further supports the hypothesis that this and other such strains are able to persist over many years in a reservoir without antimicrobial pressure, such as wild fauna or another environment yet to be defined.

## 4. Materials and Methods

### 4.1. Isolates, Culture, and DNA Extraction

The 97 isolates included in the study were gathered from the collections available at different participating laboratories, all located in the Northern and Western Europe (Supplementary Table S1). Danish isolates (n = 73) were collected between 1981 and 2016 (26 in 1981–2000 and 47 in 2011–2016), Swedish isolates (n = 2) were collected in 1996 and 2017, French isolates (n = 11) were collected in 2011–2017, Dutch isolates (n = 9) were collected in 2013, one isolate was collected from Estonia in 2011, and one was collected from Finland in 2012. The Finnish isolate was representative of a recent outbreak, out of which 9 strains were sequenced [33]; similarly, the Swedish isolate from 2017 was considered representative for recent emergence, since 2011, of *M. bovis* infections in Swedish cattle. In contrast, the isolate from 1996 was from a unique case on a single farm, and between 1996 and 2011, *M. bovis* was not diagnosed in Sweden (Anna Aspán, personal communication). For the whole genome sequence analysis, the isolates were grown in the PPLO medium supplemented as previously described [42], mycoplasma liquid (ML) medium (Mycoplasma Experience Ltd., Bletchingley, UK), or a modified Hayflick medium [43] before DNA purification using different membrane-based kits from Qiagen (QIAamp DNA minikit, DNeasy Blood and Tissue kit, Gentra Puregene kit).

### 4.2. Sequencing and in Silico Analyses

Purified genomic DNAs were sequenced on the Illumina MiSeq 2×250PE system at Wageningen Bioveterinary Research (Lelystad, The Netherlands), except for strains No. 10419 from Finland, No. 198 from Denmark, and No. 537 from Estonia, for which genomic sequences were already available [33]. Sequencing files passing the quality check were tested for non-mycoplasma DNA with the microbial classification engine “Centrifuge” [44] before being de novo assembled using SPADES [45] with k-mers 21, 33, 55, 77, 99, 127 and the “--careful” option activated. The resulting assemblies were inspected with Quast [46] and accepted with an N50 > 10,000 nucleotides. All 97 assemblies are available at the Sequence Read Archive under Bioproject accession number PRJNA602897. For comparison purposes, the sequence of the PG45 strain (RefSeq NC_014760.1) as well as two Chinese isolates from 2011 (RefSeq NC_015725.1) and 2012 (RefSeq NC_018077.1) along with a Swiss strain (RefSeq NZ_LT578453.1) were downloaded from NCBI and used as references for all phylogenetic analyses.

The contigs were annotated using the *Mycoplasma*-specific genus database from Prokka [47] using translation table 4. The rRNA genes were extracted using Barrnap.

Phylogenetic comparisons were made by calculating a whole genome average nucleotide identity (ANI) using Pyani, a Python package, as well as by using a core genome approach (500 genes of > 95% protein identity in > 99% of isolates out of 1807 genes in total) using Roary [48] and the annotations by Prokka [47]. For comparison purpose, a phylogeny based on SNP was also run with our 97 isolates using kSNP 3.0 [49] as previously described [11] with a k-mer size of 31, selected after optimization with Kchooser.

All isolates were assigned to subtypes (STs) according to the Multilocus Sequence Typing (MLST) database for *M. bovis* (https://pulmlst.org/bovis/; accessed on 07 August 2020) using the MLST package (https://github.com/tseemann/mlst) to retrieve individual allelic sequences from the genomes. The PubMLST website was developed by Keith and Jolley and is sited at the University of Oxford [50], while the scheme for *M. bovis* was developed and updated recently by Register et al. (MLST-1a legacy and MLST-1b, new) [26,28,51]. ST assignment was performed for both the old MLST-1 (MLST-1a, based on genes *adh-1*, *gltX*, *gpsA*, *gyrB*, *pta2*, *tdk*, *tkt*) and the revised MLST-1 scheme (MLST-1b, same genes, but *dnaA* in place of *adh-1*). Previously undescribed allele sequences for MLST-1a were submitted to the curator for inclusion in the database. In total, there were 1058 entries in the PubMLST database at the date of accession (August 07); 198 of these lacked information on the *adh-1* gene, and thus 890 isolates had full profiles for MLST-1a, divided into 185 different subtypes. However, there were six non-assigned complete allelic ST profiles and only a fifth of the 890 isolates originated from Europe. For MLST-1b, 628 entries had full allelic profiles divided between 136 STs, but with only 11% of the isolates from Europe. Because of the absence of the *adh-1* gene in many of the strains and the poor representation of European isolates in the PubMLST database, another MLST scheme (MLST-2) developed at APHA (Animal and Plant Health Agency) [25] was used as well to type the strains. This scheme uses another set of seven genes (*dnaA*; *metS*, *recA*, *tufA*, *atpA*, *rpoD*, *tkt*), with only *tkt* being common with MLST-1a. According to the literature, 146 isolates divided into 43 STs were retrieved, the isolates from Europe representing 63% of the total. Assigning STs according to MLST-2 involved uploading the different alleles described earlier in two studies from GenBank [27,37]. These were compiled in one text file imported to CLC Workbench (version 8.1; QiaGen Bioinformatics, QiaGen Aarhus, Denmark) and used as a local database against which the WGS assemblies were compared using BLAST. Each gene match was manually checked and assigned to the corresponding gene allele for each of the seven genes. New alleles were given numbers consecutive to the last allelic number previously described [27,37]. From these allelic numbers, new STs were arbitrary assigned to allelic combinations hitherto not described, adding 10 new allelic combinations (Table S1).

Each currently available allelic profile was downloaded from https://pubmlst.org for MLST-1a and 1b. For MLST-2, the different STs and corresponding allelic variants were retrieved from the literature, as described previously [25,41]. Seven gene categorical data were then used in further analysis and were, including the data from our strain collection, imported to GrapeTree to generate minimum spanning trees (MST) using default settings [52].

For *polC* subtyping as described by Becker et al. [24], *polC* genes were extracted with simulate_PCR [52] aligned with MUSCLE [53] and attributed a subtype (st) in comparison to those defined previously [24,31,32]. Trees were built using FastTree [54], an approximate maximum-likelihood algorithm.

All comparative genomic data were agglomerated and plotted using R v3.4.4.

Mutational hotspots associated with resistance according to Sulyok [10] were investigated by extracting the 16S and 23S rRNA genes and aligning them using MUSCLE [53] to the corresponding gene from *Escherichia coli* strain K-12 substrain MG1655 (GenBank accession number CP014225). The individual positions were then checked for mutations relative to the PG45 type strain.

The presence of resistance genes was investigated in silico by performing a BLAST analysis using the Comprehensive Antibiotic Resistance Database (CARD; https://card.mcmaster.ca/) with > 90% similarity.

### 4.3. Data Availability

The sequencing data have been deposited in the Sequencing Read Archive (SRA) under Bioproject accession number PRJNA602897.

### 4.4. Minimum Inhibitory Concentration (MIC) Assays

The MIC assays were performed in Anses laboratory of Lyon, France. Eighty-one strains of the 97 sequenced ones were selected (Supplementary Table S1), sent on dry ice to France, subcultured upon arrival for quality control, and conserved in multiple aliquots at −80 °C. Quality control was comprised of purity and species identity check by membrane filtration dot-immunoblotting test (MF-dot) and PCR [55,56].

MICs were determined using the agar dilution method on the modified PPLO agar as previously described [31]. Five antimicrobials (Sigma-Aldrich, St. Quentin Fallavier, France) were tested, corresponding to the 5 classes likely to be active on mycoplasmas: enrofloxacin (fluoroquinolones), oxytetracycline (tetracyclines), spectinomycin (aminocyclitol), florfenicol (amphenicol), tilmicosin (macrolides). In brief, 1 mL of each strain diluted to 10^4^–10^5^ CFU/mL was spotted onto agar plates containing dilutions of each antimicrobial and grown at 37 °C in 5% CO_2_ for 5 days. The MIC was defined as the lowest antimicrobial concentration causing 100% inhibition of growth after the incubation period. Only two different concentrations were used per antimicrobial. These concentrations were based on the clinical breakpoints for Pasteurellaceae as defined by the Clinical and Laboratory Standards Institute (CLSI) to be used as interpretative criteria for cattle respiratory disease (CLSI 2015). They were 2 and 8 µg/mL for oxytetracycline and florfenicol; 8 and 32 µg/mL for tilmicosin; 0.25 and 2 µg/mL for enrofloxacin, and 32 and 64 µg/mL for spectinomycin.

## 5. Conclusions

In conclusion, we compared whole genome sequences in order to analyze the genetic relatedness of 97 *M. bovis* isolates collected in Northern and Western Europe over 40 years. Sequence typing, either using the core genome or based on one or more housekeeping genes, showed a high degree of conservation in this pathogen, albeit with a recent appearance of different phylotypes in different countries between 2000 and 2008. Spread of a homogenous, clone in Denmark, which has subsequently emerged in Sweden and Finland, is also suggested. The rationale for emergence and routes of introduction of different phylotypes warrants further study. Together with the possible existence of environmental reservoirs for *M. bovis* isolates, this opens an important number of potential evolutionary scenarios for *M. bovis* sub-populations. Recent isolates share the common feature of lowered susceptibility to major antimicrobial families such as macrolides, tetracyclines, and phenicols. The preserved susceptibility to fluoroquinolones and aminoglycosides should draw our attention in the coming years, as it may evolve differently in the different sub-populations. This study highlights the importance of continuing to monitor *M. bovis* epidemiology within Europe, including other countries such as Germany, Poland, and the United Kingdom, which are important cattle producers and traders.

## Supplementary Materials

The following are available online at https://www.mdpi.com/2076-0817/9/11/875/s1. Figure S1: The genome SNP-based phylogenetic tree as generated using kSNP3.0. It was stratified by body site of isolation and country and labeled by year of isolation. The PG45 type strain from the USA as well as two Chinese isolates (including isolate Hubei-1 used as an outgroup) were included for comparison. nk, not known. Figure S2: cgMLST phylogenetic tree as generated by Roary and stratified by MLST-1a from Register et al., 2015. The Chinese Hubei-1 isolate was used as an outgroup. Figure S3: The minimum spanning tree based on MLST-1b from Register et al. (2019) prepared using GrapeTree [52] with MSTree V2. Strains from this study are color-coded. The two isolates from China included for comparison purpose are in grey. Figure S4: The minimum spanning tree based on MLST-2 from Rosales et al. (2015) prepared using GrapeTree [52] with MSTree V2 in the BioNumerics software using minimum spanning trees with default settings. Strains from this study are color-coded. The two isolates from China included for comparison purpose are in grey. Table S1: Details of the isolates used in the study with results of the genome sequencing assembly, subtyping, and antimicrobial resistance profiles.

## References

[1] Calcutt M.J., Lysnyansky I., Sachse K., Fox L.K., Nicholas R.A.J., Ayling R.D. (2018). Gap analysis of *Mycoplasma bovis* disease, diagnosis and control: An aid to identify future development requirements. Transbound. Emerg. Dis..

[2] Hale H.H., Helmboldt C.F., Plastridge W.N., Stula E.F. (1962). Bovine mastitis caused by a *Mycoplasma* species. Cornell Vet..

[3] Nicholas R.A. (2011). Bovine mycoplasmosis: Silent and deadly. Vet. Rec..

[4] Maunsell F.P., Woolums A.R., Francoz D., Rosenbusch R.F., Step D.L., Wilson D.J., Janzen E.D. (2011). *Mycoplasma bovis* infections in cattle. J. Vet. Intern. Med..

[5] Vähänikkilä N., Pohjanvirta T., Haapala V., Simojoki H., Soveri T., Browning G.F., Pelkonen S., Wawegama N.K., Autio T. (2019). Characterisation of the course of *Mycoplasma bovis* infection in naturally infected dairy herds. Vet. Microbiol..

[6] Perez-Casal J., Prysliak T., Maina T., Suleman M., Jimbo S. (2017). Status of the development of a vaccine against *Mycoplasma bovis*. Vaccine.

[7] Gautier-Bouchardon A.V. (2018). Antimicrobial Resistance in *Mycoplasma* spp.. Microbiol. Spectr..

[8] Owen J.R., Noyes N., Young A.E., Prince D.J., Blanchard P.C., Lehenbauer T.W., Aly S.S., Davis J.H., O’Rourke S.M., Abdo Z. (2017). Whole-Genome Sequencing and Concordance Between Antimicrobial Susceptibility Genotypes and Phenotypes of Bacterial Isolates Associated with Bovine Respiratory Disease. G3 Genes Genomes Genet..

[9] Parker A.M., Shukla A., House J.K., Hazelton M.S., Bosward K.L., Kokotovic B., Sheehy P.A. (2016). Genetic characterization of Australian *Mycoplasma bovis* isolates through whole genome sequencing analysis. Vet. Microbiol..

[10] Sulyok K.M., Kreizinger Z., Wehmann E., Lysnyansky I., Banyai K., Marton S., Jerzsele A., Ronai Z., Turcsanyi I., Makrai L. (2017). Mutations Associated with Decreased Susceptibility to Seven Antimicrobial Families in Field and Laboratory-Derived *Mycoplasma bovis* Strains. Antimicrob. Agents Chemother..

[11] Yair Y., Borovok I., Mikula I., Falk R., Fox L.K., Gophna U., Lysnyansky I. (2020). Genomics-based epidemiology of bovine *Mycoplasma bovis* strains in Israel. BMC Genomics.

[12] Baker S., Thomson N., Weill F.X., Holt K.E. (2018). Genomic insights into the emergence and spread of antimicrobial-resistant bacterial pathogens. Science.

[13] Register K.B., Bayles D.O., Ma H., Windeyer M.C., Perez-Casal J., Bras A.L., Suleman M., Woodbury M., Jelinski M.D., Alt D.P. (2020). Complete Genome Sequences of 16 Mycoplasma bovis Isolates from Canadian Bison and Cattle. Microbiol. Resour. Announc..

[14] Li Y., Zheng H., Liu Y., Jiang Y., Xin J., Chen W., Song Z. (2011). The complete genome sequence of *Mycoplasma bovis* strain Hubei-1. PLoS ONE.

[15] Qi J., Guo A., Cui P., Chen Y., Mustafa R., Ba X., Hu C., Bai Z., Chen X., Shi L. (2012). Comparative Geno-Plasticity Analysis of *Mycoplasma bovis* HB0801 (Chinese Isolate). PLoS ONE.

[16] Wise K.S., Calcutt M.J., Foecking M.F., Roske K., Madupu R., Methe B.A. (2011). Complete genome sequence of *Mycoplasma bovis* type strain PG45 (ATCC 25523). Infect. Immun..

[17] Josi C., Burki S., Vidal S., Dordet-Frisoni E., Citti C., Falquet L., Pilo P. (2019). Large-Scale Analysis of the *Mycoplasma bovis* Genome Identified Non-essential, Adhesion- and Virulence-Related Genes. Front. Microbiol..

[18] Feenstra A., Bisgaard Madsen E., Friis N.F., Meyling A., Ahrens P. (1991). A field study of *Mycoplasma bovis* infection in cattle. Zent. Vet. B.

[19] Arede M., Nielsen P.K., Ahmed S.S., Halasa T., Nielsen L.R., Toft N. (2016). A space-time analysis of *Mycoplasma bovis*: Bulk tank milk antibody screening results from all Danish dairy herds in 2013–2014. Acta Vet. Scand..

[20] Kusiluka L.J., Kokotovic B., Ojeniyi B., Friis N.F., Ahrens P. (2000). Genetic variations among *Mycoplasma bovis* strains isolated from Danish cattle. FEMS Microbiol. Lett..

[21] Kusiluka L.J., Ojeniyi B., Friis N.F. (2000). Increasing prevalence of *Mycoplasma bovis* in Danish cattle. Acta Vet. Scand..

[22] Petersen M.B. (2018). *Mycoplasma bovis* in Dairy Cattle: Clinical Epidemiology and Antibody Measurements for Decision Making. Ph.D. Thesis.

[23] Gulliksen S.M., Jor E., Lie K.I., Loken T., Akerstedt J., Osteras O. (2009). Respiratory infections in Norwegian dairy calves. J. Dairy Sci..

[24] Becker C.A., Thibault F.M., Arcangioli M.A., Tardy F. (2015). Loss of diversity within *Mycoplasma bovis* isolates collected in France from bovines with respiratory diseases over the last 35 years. Infect. Genet. Evol..

[25] Poumarat F., Martel J.L. (1989). In vitro antibiotic sensitivity of French strains of *Mycoplasma bovis*. Ann. Rech. Vet..

[26] Register K.B., Thole L., Rosenbush R.F., Minion F.C. (2015). Multilocus sequence typing of *Mycoplasma bovis* reveals host-specific genotypes in cattle versus bison. Vet. Microbiol..

[27] Rosales R.S., Churchward C.P., Schnee C., Sachse K., Lysnyansky I., Catania S., Iob L., Ayling R.D., Nicholas R.A. (2015). Global multilocus sequence typing analysis of *Mycoplasma bovis* isolates reveals two main population clusters. J. Clin. Microbiol..

[28] Register K.B., Lysnyansky I., Jelinski M.D., Boatwright W.D., Waldner M., Bayles D.O., Pilo P., Alt D.P. (2020). Comparison of Two Multilocus Sequence Typing Schemes for *Mycoplasma bovis* and Revision of the PubMLST Reference Method. J. Clin. Microbiol..

[29] Shakya M., Ahmed S.A., Davenport K.W., Flynn M.C., Lo C.C., Chain P.S.G. (2020). Standardized phylogenetic and molecular evolutionary analysis applied to species across the microbial tree of life. Sci. Rep..

[30] CLSI (2015). Performance Standards for Antimicrobial Disk and Dilution Susceptibility Tests for Bacteria Isolated from Animals.

[31] Khalil D., Becker C.A., Tardy F. (2016). Alterations in the Quinolone Resistance-Determining Regions and Fluoroquinolone Resistance in Clinical Isolates and Laboratory-Derived Mutants of *Mycoplasma bovis*: Not. All Genotypes May Be Equal. Appl. Environ. Microbiol..

[32] Khalil D., Becker C.A.M., Tardy F. (2017). Monitoring the Decrease in Susceptibility to Ribosomal RNAs Targeting Antimicrobials and Its Molecular Basis in Clinical *Mycoplasma bovis* Isolates over Time. Microb. Drug. Resist..

[33] Haapala V., Pohjanvirta T., Vähänikkilä N., Halkilahti J., Simonen H., Pelkonen S., Soveri T., Simojoki H., Autio T. (2018). Semen as a source of *Mycoplasma bovis* mastitis in dairy herds. Vet. Microbiol..

[34] Heuvelink A., Reugebrink C., Mars J. (2016). Antimicrobial susceptibility of *Mycoplasma bovis* isolates from veal calves and dairy cattle in the Netherlands. Vet. Microbiol..

[35] Petersen M.B., Krogh K., Nielsen L.R. (2016). Factors associated with variation in bulk tank milk *Mycoplasma bovis* antibody-ELISA results in dairy herds. J. Dairy Sci..

[36] Burki S., Spergser J., Bodmer M., Pilo P. (2016). A dominant lineage of *Mycoplasma bovis* is associated with an increased number of severe mastitis cases in cattle. Vet. Microbiol..

[37] Lysnyansky I., Freed M., Rosales R.S., Mikula I., Khateb N., Gerchman I., Straten M.v., Levisohn S. (2016). An overview of *Mycoplasma bovis* mastitis in Israel (2004–2014). Vet. J..

[38] Becker C.A.M., Ambroset C., Huleux A., Vialatte A., Colin A., Tricot A., Arcangioli M.A., Tardy F. (2020). Monitoring *Mycoplasma bovis* Diversity and Antimicrobial Susceptibility in Calf Feedlots Undergoing a Respiratory Disease Outbreak. Pathogens.

[39] Abu-Amero K.K., Abu-Groun E.A., Halablab M.A., Miles R.J. (2000). Kinetics and distribution of alcohol oxidising activity in *Acholeplasma* and *Mycoplasma* species. FEMS Microbiol. Lett..

[40] Josi C., Burki S., Stojiljkovic A., Wellnitz O., Stoffel M.H., Pilo P. (2018). Bovine Epithelial in vitro Infection Models for *Mycoplasma bovis*. Front. Cell Infect. Microbiol..

[41] Gautier-Bouchardon A.V., Ferre S., Le Grand D., Paoli A., Gay E., Poumarat F. (2014). Overall decrease in the susceptibility of *Mycoplasma bovis* to antimicrobials over the past 30 years in France. PLoS ONE.

[42] Poumarat F., Longchambon D., Martel J.L. (1992). Application of dot immunobinding on membrane filtration (MF dot) to the study of relationships within “*M. mycoides* cluster” and within “glucose and arginine-negative cluster” of ruminant mycoplasmas. Vet. Microbiol..

[43] Kobisch M., Friis N.F. (1996). Swine mycoplasmoses. Rev. Sci. Tech..

[44] Kim D., Song L., Breitwieser F.P., Salzberg S.L. (2016). Centrifuge: Rapid and sensitive classification of metagenomic sequences. Genome Res..

[45] Bankevich A., Nurk S., Antipov D., Gurevich A.A., Dvorkin M., Kulikov A.S., Lesin V.M., Nikolenko S.I., Pham S., Prjibelski A.D. (2012). SPAdes: A new genome assembly algorithm and its applications to single-cell sequencing. J. Comput. Biol..

[46] Gurevich A., Saveliev V., Vyahhi N., Tesler G. (2013). QUAST: Quality assessment tool for genome assemblies. Bioinformatics.

[47] Seemann T. (2014). Prokka: Rapid prokaryotic genome annotation. Bioinformatics.

[48] Page A.J., Cummins C.A., Hunt M., Wong V.K., Reuter S., Holden M.T., Fookes M., Falush D., Keane J.A., Parkhill J. (2015). Roary: Rapid large-scale prokaryote pan genome analysis. Bioinformatics.

[49] Gardner S.N., Slezak T., Hall B.G. (2015). kSNP3.0: SNP detection and phylogenetic analysis of genomes without genome alignment or reference genome. Bioinformatics.

[50] Jolley K.A., Bray J.E., Maiden M.C.J. (2018). Open-access bacterial population genomics: BIGSdb software, the PubMLST.org website and their applications. Wellcome Open Res..

[51] Register K.B., Jelinski M.D., Waldner M., Boatwright W.D., Anderson T.K., Hunter D.L., Hamilton R.G., Burrage P., Shury T., Bildfell R. (2019). Comparison of multilocus sequence types found among North. American isolates of *Mycoplasma bovis* from cattle, bison, and deer, 2007–2017. J. Vet. Diagn. Investig..

[52] Gardner S.N., Slezak T. (2014). Simulate_PCR for amplicon prediction and annotation from multiplex, degenerate primers and probes. BMC Bioinform..

[53] Edgar R.C. (2004). C. MUSCLE: Multiple sequence alignment with high accuracy and high throughput. Nucleic Acids Res..

[54] Price M.N., Dehal P.S., Arkin A.P. (2010). FastTree 2–Approximately maximum-likelihood trees for large alignments. PLoS ONE.

[55] Marenda M.S., Sagne E., Poumarat F., Citti C. (2005). Suppression subtractive hybridization as a basis to assess *Mycoplasma agalactiae* and *Mycoplasma bovis* genomic diversity and species-specific sequences. Microbiology.

[56] Poumarat F., Perrin B., Longchambon D. (1991). Identification of ruminant mycoplasmas by dot immunobinding on membrane filtration (MF dot). Vet. Microbiol..

