# Supplementary figures and images for: Mycoplasma bovis in Nordic European Countries: Emergence and Dominance of a New Clone
Source: Pathogens. 2020 Oct 23;9(11):875. doi: 10.3390/pathogens9110875 (PMC7716209; doi:10.3390/pathogens9110875)

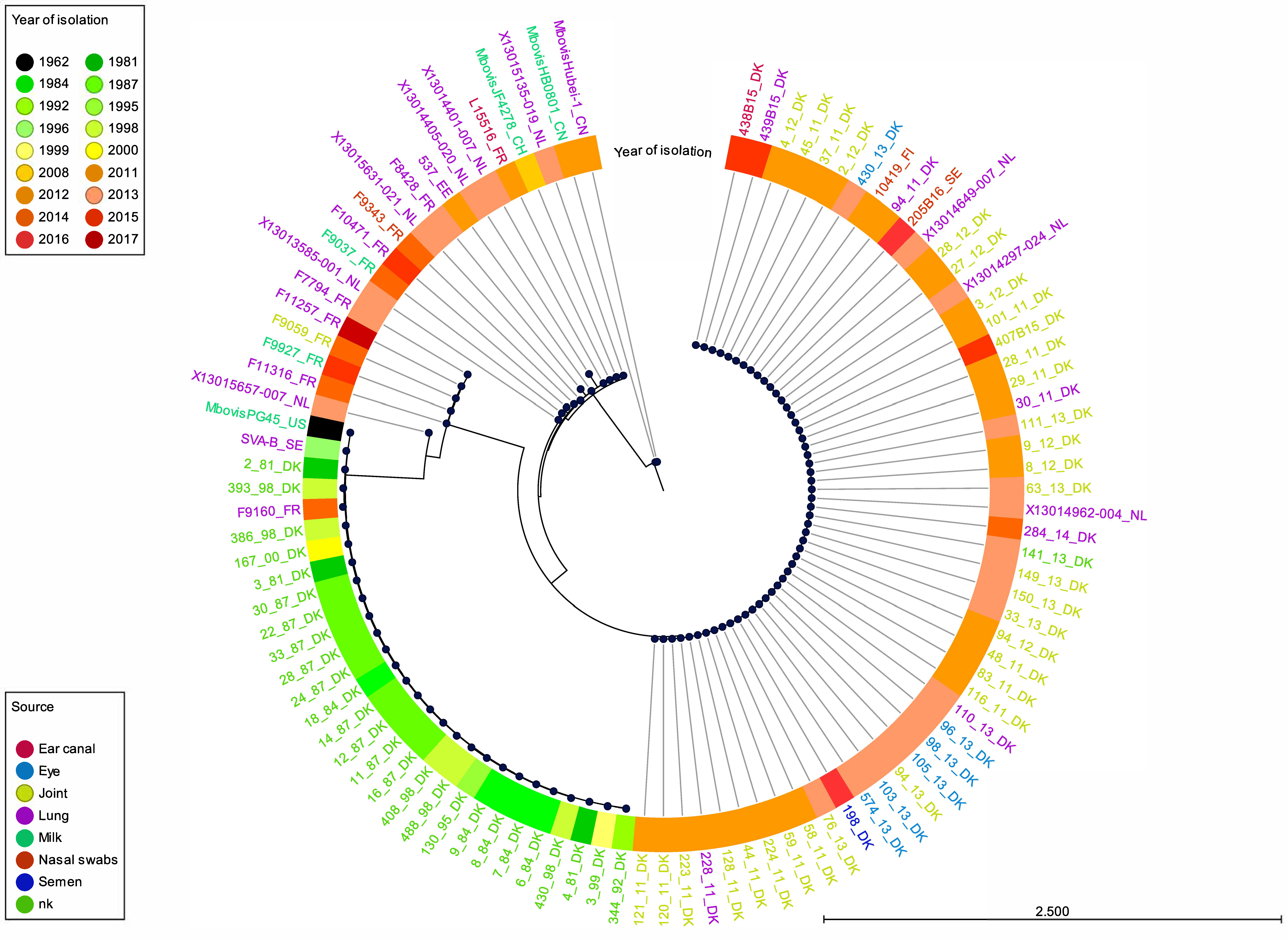

Supplement: Supplementary file 1 [file pathogens-09-00875-s001.zip › Supplementary-Final Version/FigS1_kSNPhylo_070920.png]

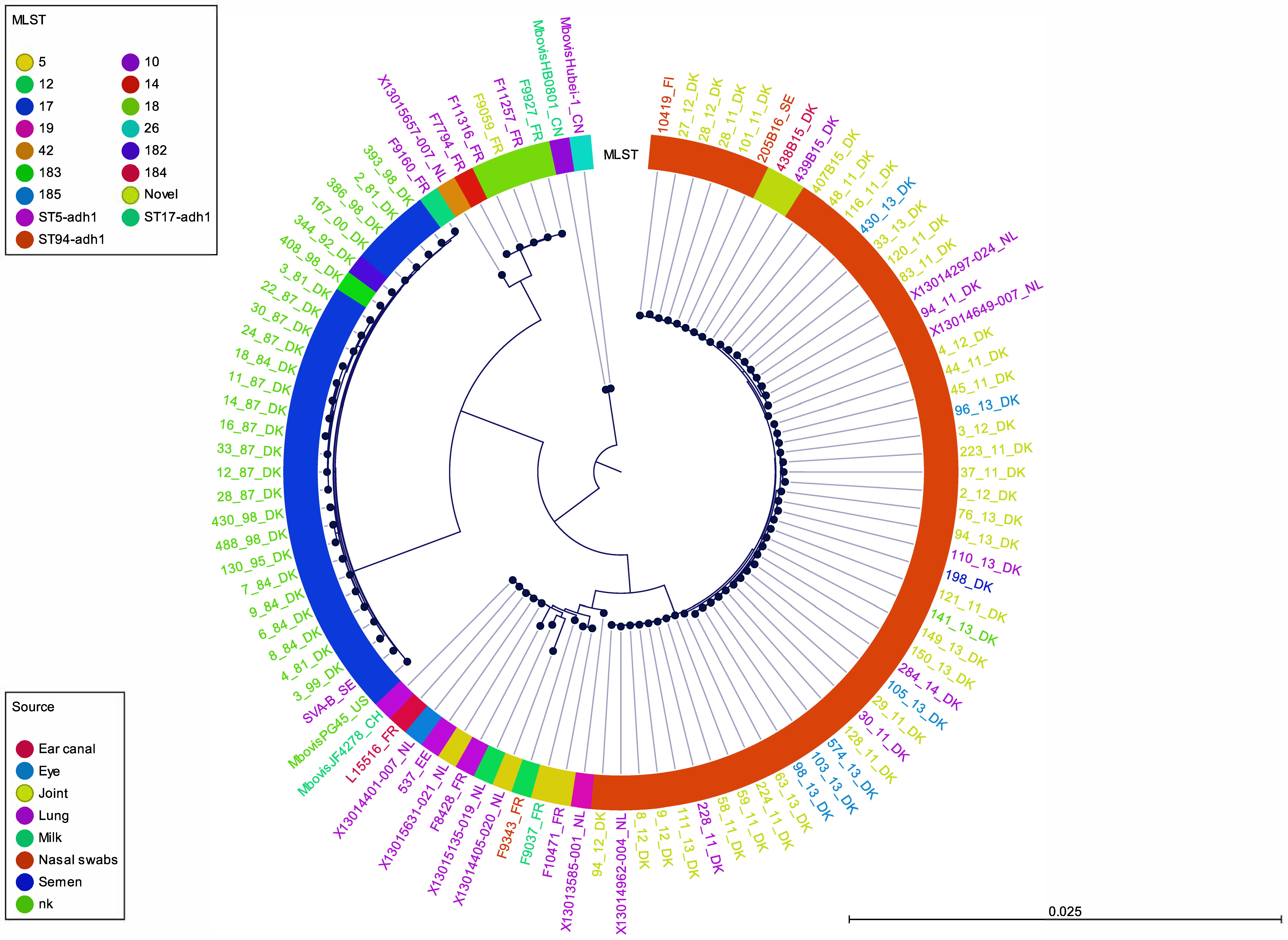

Supplement: Supplementary file 1 [file pathogens-09-00875-s001.zip › Supplementary-Final Version/FigS2_REV7oct2020.png]

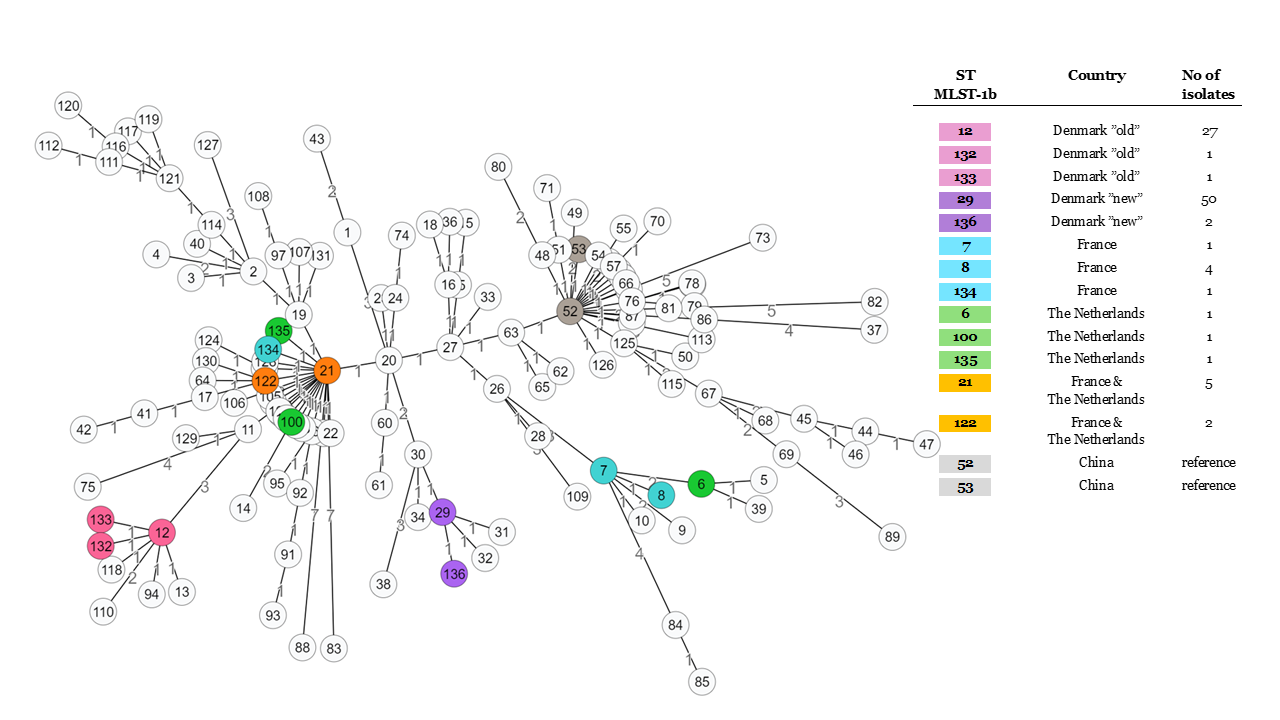

Supplement: Supplementary file 1 [file pathogens-09-00875-s001.zip › Supplementary-Final Version/FigS3_REV7oct_MLST_1b.png]

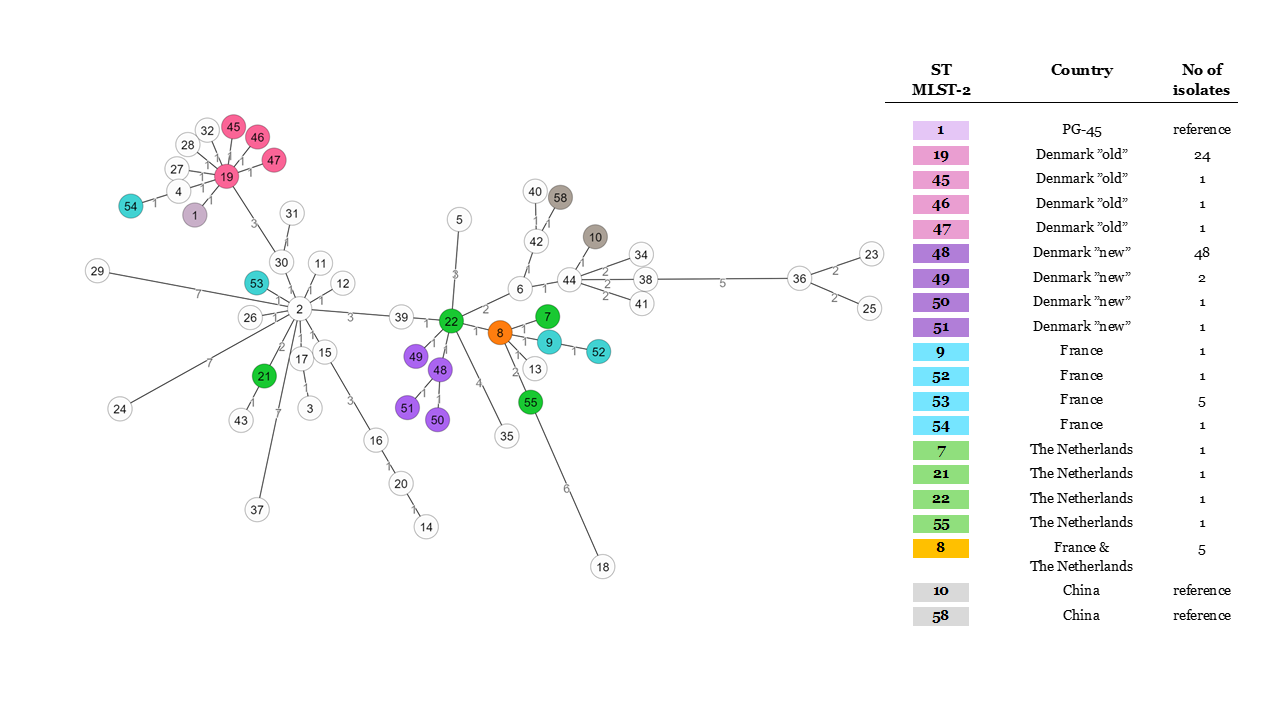

Supplement: Supplementary file 1 [file pathogens-09-00875-s001.zip › Supplementary-Final Version/FigS4_REV7octMLST_2.png]
